# Supplementary material for: Characterization of 18F-PM-PBB3 (18F-APN-1607) Uptake in the rTg4510 Mouse Model of Tauopathy
Source: Molecules. 2020 Apr 10;25(7):1750. doi: 10.3390/molecules25071750 (PMC7181044; doi:10.3390/molecules25071750)
Supplement: Supplementary file 1 [file molecules-25-01750-s001.pdf]

molecules

Supplemental Materials

S.1 Radiosynthesis of ^{18}F-PM-PBB3

The [^{18}F] fluoride was trapped on an Accel Plus QMA (preconditioned with 10 mL of 1M K_2CO_3 and 60 ml of purified water), and was then eluted to the reaction vessel using a 1 ml mixture of K_2CO_3 (3.6 mg)/Kryptofix2.2.2(7.1 mg) in a 5:2 (*v*/*v*) solution of acetonitrile /water. The [K/Kryptofix]+^{18}F complex was dried at 85 ℃ for 2 min, and then placed under a vacuum and helium flow at 100 ℃ for 7 min. Further drying was carried out by forming an azeotrope with 0.5 mL of anhydrous acetonitrile at 100 ℃ for 3 min under a vacuum and helium flow. A solution of the precursor of ^{18}F-PM-PBB3 (2 mg) in1 mL of DMSO was added on the air-cooled dried residue in the reaction vessel, and was then heated to 110℃ for 10 min to produce fluorinated intermediate, N-Boc-^{18}F-PM-PBB3. The protection group of the fluorinated intermediate was hydrolyzed using HCl (3.33 N;0.8 mL) and heated at 90 ℃ for 4 min. After cooling to 50 ℃, the reaction mixture was neutralized using an 8 mL portion of an aqueous solution containing 10% NaOH and 4% ammonium acetate.

The solution was loaded onto the first Sep-Pak C18 light cartridge. The ^{18}F-luoride, salts and most polar species were removed from the cartridge by washing with 10 ml of sterile water for injection. The crude ^{18}F-PM-PBB3 was eluted with 1.2 mL of acetonitrile into the HPLC reservoir and was further diluted with 1 mL of 0.1 M ammonium acetate solution. The mixture in the HPLC reservoir was loaded to a 2 mL loop and then injected to a semi-preparative column (Atlantis prep T3 5um, 10 x 150 mm C18 column Waters.co) equipped on a Tracerlab fxn pro synthesizer HPLC system for purification. The HPLC column was eluted with a mixture of acetonitrile and 50 mM ammonium acetate (200:300) containing 0.5% (*w*/*v*) sodium ascorbate, at a flow rate of 5.0 mL/min. The peak fraction corresponding to the non-radioactive standard ^{19}F-PM-PBB3 (approximately at 12 min) was collected in the round-bottom flask in which the eluate was diluted with 20 mL of sterile water for the injection containing 0.5% (*w*/*v*) sodium ascorbate.

The solution was passed through the C18 Sep-Pak Plus cartridge, which was further washed with 16 ml of sterile water for the injection containing 0.5% (*w*/*v*) sodium ascorbate. ^{18}F-PM-PBB3 was eluted using 1.5 mL of ethanol into the product vessel preloaded with 3.0 mL of normal saline containing 0.5% (*w*/*v*) sodium ascorbate. We rinsed the C18 Sep-Pak Plus cartridge with 4.0 mL of normal saline containing 0.5% (*w*/*v*) sodium ascorbate in the product vessel and then transferred the solution through a sterile Millex GV filter into a sterile 20-mL final product vial preloaded with 7.0 mL of normal saline containing 0.5% (*w*/*v*) sodium ascorbate. The final drug product of ^{18}F-PM-PBB3 was dispensed, in a Class A (PIC/S GMP) hot cell, into a sterile pyrogen-free glass vial by passing through a 0.22-mm sterile Dualex filter attached to the vial. The integrity of the filter was immediately confirmed by bubble-point test when the sterile filtration was completed. The total activity of ^{18}F-PM-PBB3 at the end of synthesis (EOS) was 9.3–18.5 GBq.

The radiochemical purity of ^{18}F-PM-PBB3 was determined using a HPLC system equipped with a C18 column (XBridge C18, 5 μm, 4.6 × 250 mm PN: 186003117; Waters), which was eluted with a mobile phase MeOH and 5mM NH4OAc in HPLC grade water at a flow rate of 1mL/min in the gradient condition shown in Table S1. The absorbance of 365 nm and the radioactivity in the eluate flow were monitored with a diode array detector and a flow count detector, respectively. Residual solvents (ethanol, acetonitrile, and DMSO) were quantified using a gas chromatograph system equipped with an FID detector and a Varian Capillary Column CP-Wax 52CB (30.0 m × ID 530 μm × OD 1 μm; PN#CP8738)).

Table 1. HPLC gradient condition for the radiochemical purity analysis of ^{18}F-PM-PBB3.

**Time (min)**	**MeOH (per cent *v*/*v*)**	**5mM NH_4OAc (per cent *v*/*v*)**
0	70	30
8	70	30
15	95	5
17	95	5
17.5	70	30
20	70	30
Post run time	3 min	

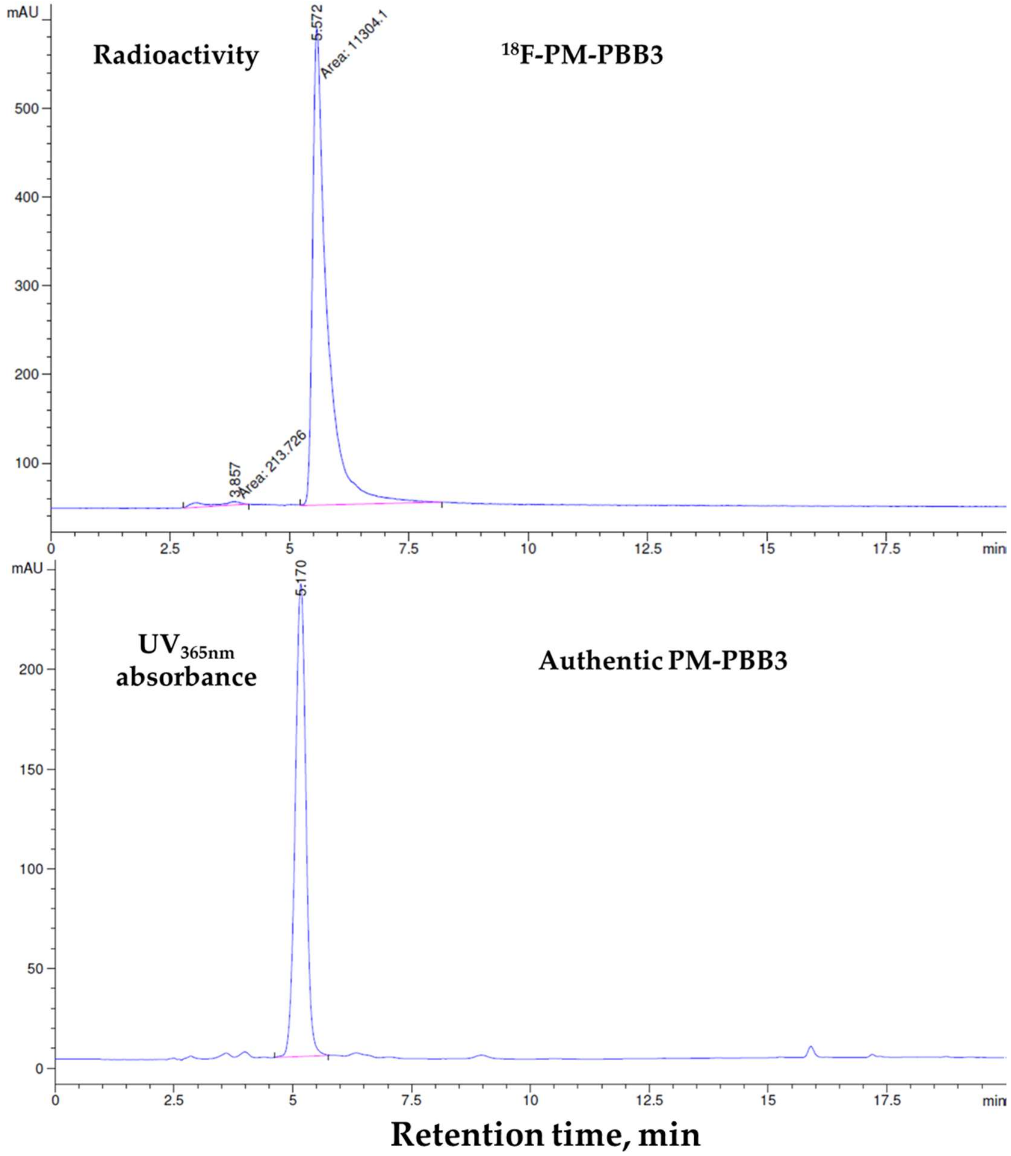

Figure 1. HPLC Profile of ^{18}F-PM-PBB3.

S. 2 PET Acquisition.

All animal PET studies were performed using an Inveon PET scanner at Chang Gung Memorial Hospital. The Inveon PET is a commercial PET scanner (Siemens Medical Solutions USA, Inc.) designed based on the microPET Focus system with improved sensitivity. The transaxial and axial fields of view are 10 and 12.7 cm, respectively. All data were acquired at the coincidence timing window of 3.5 ns and the energy window of 350–650 keV, and corrected for radioactive decay, random coincidence counts, and dead-time losses. The peak system sensitivity is 6.8% at the center of the field of view (CFOV). The reconstructed-image and volumetric resolution of the Inveon PET are 1.4 mm in full at half maximum and 2.5 μL close to the CFOV, respectively.

For the PET study, rTg4510 mice were deprived of food overnight at least 8 h before radiotracer injection. Animals had constant access to drinking water. Warming was started 30 min before radiotracer injection and continued throughout the uptake and imaging period under a tungsten lamp. The scanning room temperature was controlled at 24 °C at all times. The radiotracer was injected intravenously into the tail vein of each mouse after a short (5 min) isoflurane (1% in 100% oxygen) anesthesia period.

S. 3 Acquisition Protocol.

For the pilot study, dynamic PET data for each animal was acquired for 90 minutes with time frames (18 × 10 sec, 4 × 30 sec, 5 × 1 min, 10 × 5 min, 3 × 10 min) starting right after radiotracer injection with the animal in a prone bed position. List mode image data were acquired and rebinned into 2D sinograms using the Fourier rebinning method. All PET images were reconstructed using the 2D OSEM method (4 iterations and 16 subsets) without attenuation and scatter corrections. There were a total of 159 slices of reconstructed images, each having a matrix size of 128 × 128. The resulting slice thickness and pixel size were 0.0796 and 0.0861 cm, respectively. To derive a system calibration factor between the known activity from the dose calibrator and the mean voxel values from the animal PET reconstruction, a 3.0-cm cylinder phantom filled with a known concentration of 18F was imaged and processed previously.

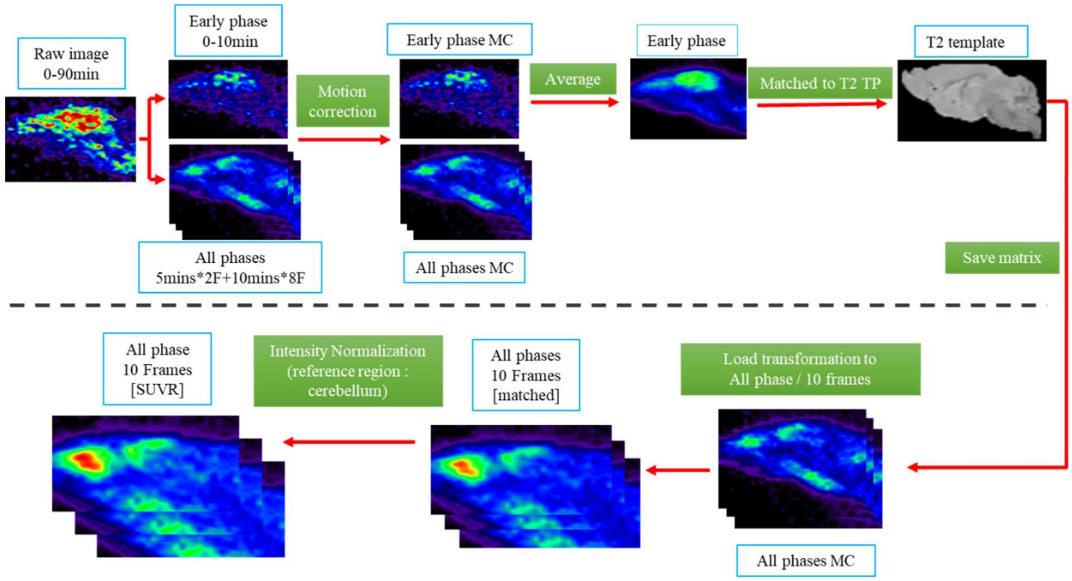

Figure 2. The procedure for creating the PET imaging template for each animal for further PET image analysis applied a rigid match with the MNI MRI T2 template.

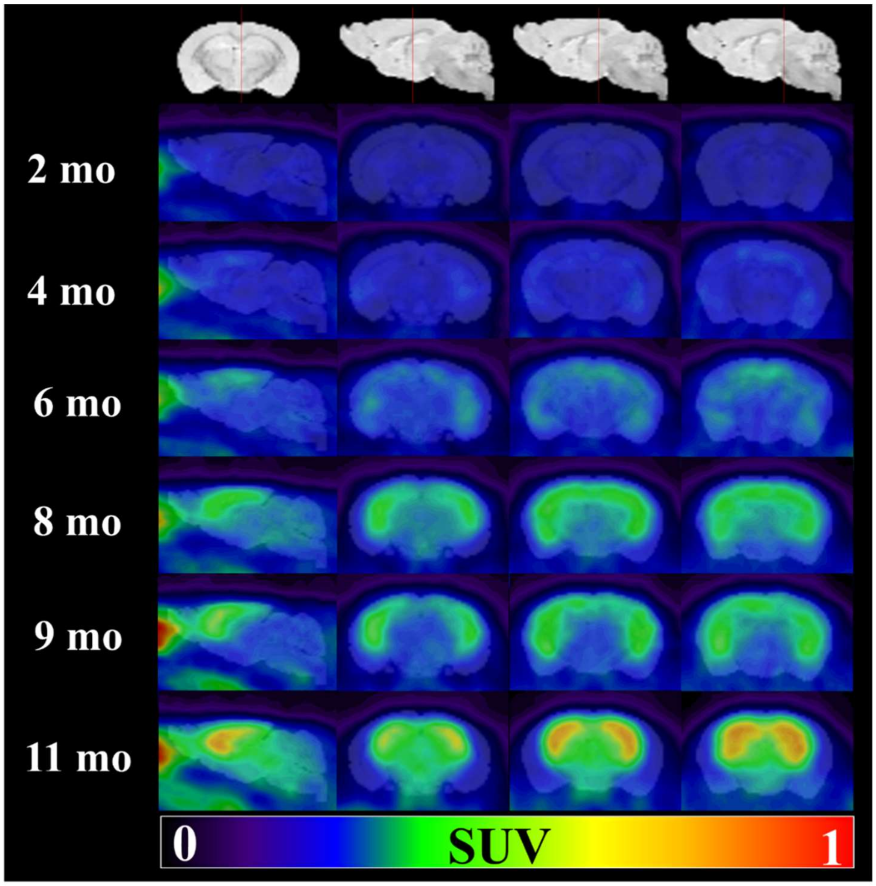

Figure 3. Representative ^{18}F-PM-PBB3 PET images of different animal groups.
